# Soft integration of a neural cells network and bionic interfaces

**DOI:** 10.3389/fbioe.2022.950235

**Published:** 2022-09-29

**Authors:** Jixiang Zhang, Ting Wang, Yixin Zhang, Pengyu Lu, Neng Shi, Weiran Zhu, Chenglong Cai, Nongyue He

**Affiliations:** ^1^ State Key Laboratory of Bioelectronics, National Demonstration Centre for Experimental Biomedical Engineering Education, School of Biological Science and Medical Engineering, Southeast University, Nanjing, China; ^2^ Southeast University Jiangbei New Area Innovation Institute, Nanjing, China; ^3^ SceneRay Co., Ltd., Suzhou, China

**Keywords:** brain–computer interface, neural network, glia cell, biointerface, nanoparticle, neuromodulation, bioinspired design

## Abstract

Both glial cells and neurons can be considered basic computational units in neural networks, and the brain–computer interface (BCI) can play a role in awakening the latency portion and being sensitive to positive feedback through learning. However, high-quality information gained from BCI requires invasive approaches such as microelectrodes implanted under the endocranium. As a hard foreign object in the aqueous microenvironment, the soft cerebral cortex’s chronic inflammation state and scar tissue appear subsequently. To avoid the obvious defects caused by hard electrodes, this review focuses on the bioinspired neural interface, guiding and optimizing the implant system for better biocompatibility and accuracy. At the same time, the bionic techniques of signal reception and transmission interfaces are summarized and the structural units with functions similar to nerve cells are introduced. Multiple electrical and electromagnetic transmissions, regulating the secretion of neuromodulators or neurotransmitters *via* nanofluidic channels, have been flexibly applied. The accurate regulation of neural networks from the nanoscale to the cellular reconstruction of protein pathways will make BCI the extension of the brain.

## Introduction

The ecosystem of a neural network is composed of neurons and glial cells immersed in an extracellular matrix, and they are extensively connected in the brain. The nucleus, ganglia, and cerebral cortex form an interconnected network (Bostan and Strick, 2018; Greene et al., 2020). Changes in the organization of functional connections allow information to be analyzed and processed, and thus the neural network appears in the brain. The generation of neural networks and signal transmission processes are closely related to electrical and chemical signals. Consciousness and intelligence were considered to be hidden behind the largely associated and well-organized information networks, which have dynamic parallel storage and analysis functions. To better understand the neural network and cure brain diseases, integrated brain–computer interfaces (BCIs) to perform afferent and efferent functions were developed. BCIs could not only replace injured sense organs and improve motor debilities, such as stroke rehabilitation (Biasiucci et al., 2018) that translate neural intentions into movement and are helpful in amyotrophic lateral sclerosis, but also can modulate emotions and memories and monitor brain health, such as tumorigenesis and stroke risks. The BCI plays a core role in mapping neural signals. However, hard BCI brain electrodes can cause a lot of damage to brain tissue, such as scars and inflammation. In this study, we will discuss the ways to realize the bionic neural network information interface, overcoming the long-term defects of scar tissue and losing control of lacking environmental interactions.

Noninvasive BCI systems based on electroencephalogram (EEG) signals will be a strategy to overcome the long-term defects of scar tissue and inflammation. Because of their penetration of the skull through various energy and information patterns, the brain electrode contact interface can be exploited on the knowledge of the original brain physiology. However, their less precision to interact with brain networks led to unsuccessfully applied for precisely timed control tasks. Other noninvasive detections are based on the nutrients sensitive to neurons and blood flow to oxy and deoxygenated hemoglobin; functional magnetic resonance imaging (fMRI) as well as functional near-infrared spectroscopy (fNIRS) are favorable neuroimaging modalities. They are commonly used for confirming brain areas of a functional population of neurons that have coordinated activities in noninvasive BCI systems (Golub et al., 2018).

A more accurate way to effectively find targeted nerve nuclei and observe the patient’s response to stimulation was combined using deep brain stimulation and transcranial magnetic stimulation. TMS uses a pulsed magnetic field to influence the membrane potential of nerve cells, causing them to produce induced currents, affecting brain metabolism and electrical activity. DBS records the characteristics of different depths and orbits of the brain to find targeted nerve nuclei and observe the patient’s response to stimulation. This EEG scan not only allows the surgeon to pinpoint the right target to place the electrode but it can also serve as a source of energy and information for intracranial implantable therapy. The neural population dynamics based on the aforementioned technology inform the implantation of BCI systems in the functional units of the brain’s nucleus and subregions (Trautmann et al., 2021). After that, learning models can effectively meet the demand for the large amount of homogeneous training data (Khalil et al., 2022).

The materials such as intracerebral nano-implants for the brain–computer interface mean a type of information penetration technology. The skull can be penetrated by optical and magnetic approaches, and the afferent and efferent information of the neural network can be realized by the information conversion from the implant to control the action potential of neurons. Nanomaterials have unique characteristics such as similar dimensions to neural functional structures and higher spatiotemporal precision for seamless integration (Yang et al., 2021), biocompatibility, and electrically conductive, as well as energy and information conversion (Chen et al., 2018; Liu et al., 2021). However, its safety to the nervous system remains to be evaluated (Xu et al., 2021). In detail, the instantaneous neuron voltage change caused by action potential is determined by the ion concentration on both sides of the plasma membrane, which carries information by spike potential frequency and can be simplified and presented digitally as the highest fidelity of extra-neural messages. To maintain ionic gradients and then achieve interface digitalization, an energy-consuming process, a constant supply of glucose and oxygen is required for neurons. To achieve digitization, it is important to maintain nutrient supply or to regulate interface nutrient supply because of the maintenance of ion charge gradients during this process.

Apart from the biphase actions of nanoparticles for neurons, glial cells’ chemotaxis (Rostene et al., 2007), tissue damage, and inflammation limit the BCI’s long-term stability, especially in tracking single neuronal activity (Gu et al., 2021). A biocompatible and perdurable BCI requires chemical, mechanical, and physiological properties of the interface to match the structure and function of the neural network. When it comes to the modifications of the arrays, probes and nanoparticles systems, functional groups, and peptides, modular mechanical, neuromorphic, roughness, and density are supposed to be considered to live with the brain network’s cell potential difference of the brain network, hydrophobic lipid membrane, and aqueous environment (Terstappen et al., 2021). Only the integration of neural networks and artificial BCIs can form a long-term brain–computer interface system through the influence of synapses and the reduction of inflammatory responses and chemotaxis of glial cells, such as mimicking synaptic release and enzyme reactions, reducing inflammatory responses, and antagonizing glial chemotaxis. The problem can be ameliorated with anti-inflammatory hormones, and some protein molecules such as enzymes and regulatory factors that have pharmacological effects also need to be considered when connected to neural networks.

**FIGURE 1:**
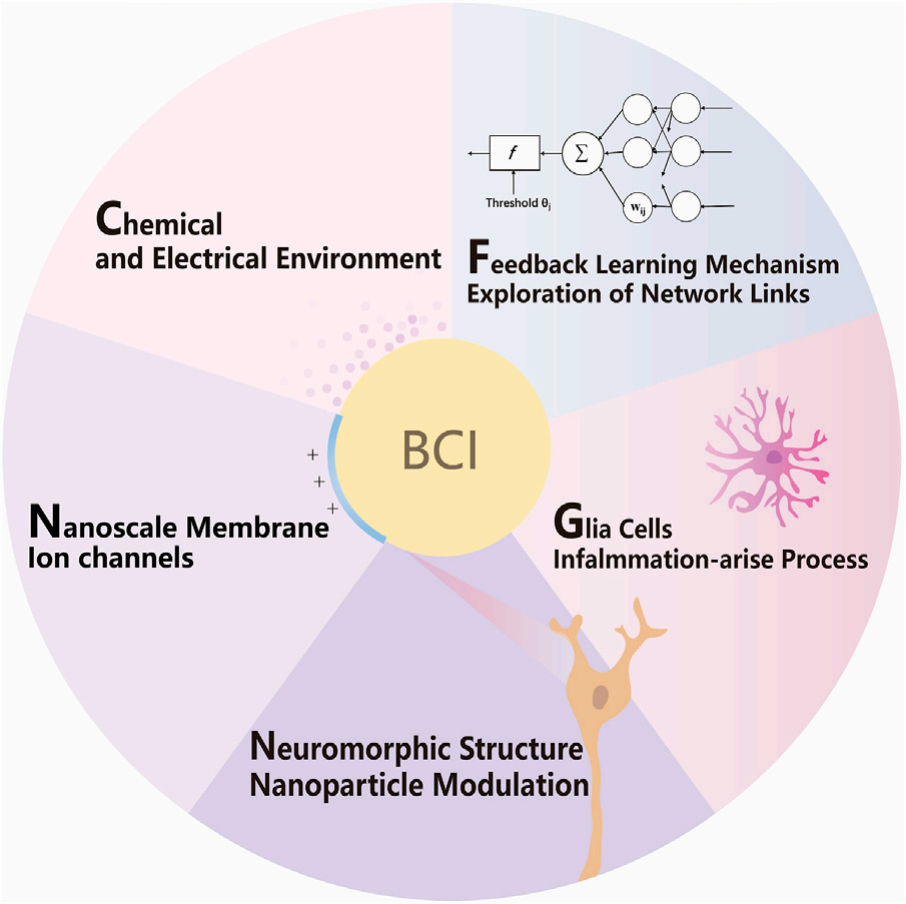
Schematic of strategies for molecular, cellular, and network modulation of BCI.

Internal information exchanges also stimulate the fusion of the interface and lead to an increase in functional connectivity such as coupling to neurotransmitters or functional electrical stimulation (Biasiucci et al., 2018). The brain is not just layers of cell islands but an intricate relation in different aspects of the nervous system and the overall body. The network receives various kinds of message and constantly modulates itself. The feedback reward system generally exists in the brain for behavior encouragement, strengthened by the release of dopamine. In addition, glutamate is the most common neurotransmitter in neural networks, as a rapid excitatory synaptic connection between two neurons and slowly shaping neuroplasticity related to neuronal development, such as learning and memory. These substances have a simple biosynthetic process, release frequently, and diffuse across the synaptic cleft. Receptors selectively bind to these chemicals and function as changing postsynaptic neurons in either excitation or inhibitory way, depolarizing or repolarizing, respectively. These are the zero and ones that have inner similarities to electronic signals, and they will be influenced by the features and chemical environment of neuron interfaces, nanoscale membrane and ion channels, nanoparticle interfaces, glial cells, inflammation-arise process, feedback learning mechanism, and exploration of network links. Accordingly, five aspects of neural network and interface system integration are proposed to promote digital connection to the brain in the following (Figure 1).

As shown in Figure 1, by better fitting bionic interfaces and modulating glial inflammatory responses, researchers can achieve functional manipulation and information reception on single-cell functional systems, thus achieving the purpose of brain–computer interaction bi-directionally and treatment of neurological diseases. The key points are as follows: features and chemical environment of neuron interfaces, nanoscale membrane and ion channels, nanoparticle interfaces, glial cells, inflammation-arise process, feedback learning mechanism, and exploration of network links; a detailed description will be expanded one after another.

## Features and chemical environment of neuron interfaces

Stabilization inside and outside the cell is based on concentration gradients and ionic gradients, which are influenced by chemical signals, electrical signals, and nutrition. The homeostasis of the neural environment meets the high metabolic demand due to the blood–brain barrier (BBB) and neurovascular coupling, performing selective substance transport; the latter make sure the activated neural regions with adequate nutrients and acidic metabolites are removed by increased blood flow (Kaplan et al., 2020). In addition, a stable and nutrient-rich internal environment, signal stimulation, and glial microenvironment regulation are the key links to the neuron’s survival, especially near the critical interface regions. For example, as a neurotransmitter, dopamine could contribute to the reward system by affecting synaptic conditioning and plasticity, and thus regulate learning, motion control, and moods. At this point, regulation of synaptic weights has been achieved utilizing the neuromorphic interface design and simulation of the dopamine recycle in the synaptic cleft (Keene et al., 2020).

For electrodes, cellular metabolism is associated with the bioelectrochemical process, and certain molecular structures significantly affect electron transfer and absorbed forms. Electrons are transferred to one-electron molecule receptors, such as cytochrome C, to a macroscopic electrode (Bollella & Katz, 2020). The electrostatic attraction of protein residues to the electrode surface with opposite polarity resulted in the fixation of proteins in an electrically active orientation, which promoted direct electron transfer between the redox center and the electrode (Bollella & Katz, 2020). Neuronal morphology electronics with better biocompatibility and relatively low mechanical modulus can imitate subcellular structural features and mechanical properties such as synapse formation and membrane potential. For example, the artificial synapse takes advantage of the dopamine feedback to strengthen the mechanism. The exocytose dopamine at the presynaptic end can be oxidized locally at the postsynaptic gate electrode, resulting in the charge state alteration of the gate electrode and inducing ion flow in the aqueous electrolyte (Yang et al., 2019; Keene et al., 2020; Wei et al., 2021).

As advanced electrodes are much preferred to abandon the hard electrode mode, hydrogels can be used as BCI substrates to simulate the brain’s internal environment. Similar mechanical, chemical, and electrical properties effectively reduce the inflammatory response of tissue damage and glial cell aggregation, which is suitable for long-term invasive BMI. The bioadhesive ensures seamless contact with the neuronal network and thus serve as interface materials recording high-quality local field potentials and individual spikes, colocating transmitting laser waveguide stimulation, tin-made microelectrode recording, and fluidic channels such as optic fibers, ionic, electric, and chemical regulation conductor hybrid. In addition, the hardness of the hydrogel varies at different temperatures or hydrate levels, which allows for different shapes and hardness during and after the implantation operation (Sheng et al., 2019; Park et al., 2021). Such BMI can be manufactured by a self-setting process of hydrogel on a microelectrode array (Wang et al., 2022) or the brain tissue surface. Neural interface hydrogels are available in a variety of formulations. Polyethylene glycol and artificial cerebrospinal fluid hydrogels are stretchable, transparent, and ionic conductors and can be made into both ionotropic and optical fibers. In addition, it is reported that polyethylene glycol also has been used to culture neurons for providing a similar environment (Sheng et al., 2019). Furthermore, hydrophilic ultra-small conductive polymer nanoparticles were introduced into the carrageenan–polydopamine–polyacrylamide network. In addition, multifunctional sensing and actuation hydrogel matrix BCI systems are mixed with multimaterial fibers with adaptive bending stiffness adjusted by hydration states. These hydrogel-based brain–computer interfaces are expected to be able to conduct light, electricity, and molecules all at once (Park et al., 2021). Meanwhile, for high conductivity, extensibility, low modulus, and excellent mechanical and electrical stability, catechol functional groups were introduced into the hydrogel to avoid body rejection reaction (Wang et al., 2022), at the same time poly (3,4-ethylene dioxythiophene), poly (styrene sulfonate), or poly (ethylenimine) and partially reduced graphene oxide surface are used for the conductive hydrogel (van de Burgt et al., 2017; Lee et al., 2019; Keene et al., 2020).

Furthermore, widely applied chemogenetics and optogenetics with high cell-type specificity regulating the ion channel structure and potential polarity change can use polyacrylamide hydrogel as optical fibers, performing colocating the site of stimulation and recording. For example, hydrogel, as a soft material with lower glial scars and immune response, could simultaneously stimulate and record neurons utilizing optogenetics (Sheng et al., 2019). Furthermore, cellular-scale semiconductor devices, consisting of light-emitting diodes, actuators, sensors, and silicon devices, can operate precisely in actuation and sensing (Kim et al., 2013).

## Nanoscale membrane and ion channels

From the abundant cerebral sulcus to the dendritic spines, certain thresholds are distributed over a wide range of neuronal cell projections. The thresholds on the membrane surface allow the brain to have unique functions. Establishing connections for synaptic-level fusion is the goal to achieve brain–machine interactions. In the change of energy and matter, the membrane is the center of information that can ion flow, electric field change, and other neuron-recognizable information to stimulate the membrane structure, and thus generate artificial neural pathways. Cell membrane regions are basic neural network units for analyzing and transforming information logically, where specialized, and are mainly composed of ion channels and receptor proteins. Key chemical microenvironment, mobility, and ionic gradients are relevant to redox processes, nutrient conversion, enzyme catalysis, glial cell guidance, and ion channels.

The large extension of the neuronal plasma membrane determines polarity conduction and signal processing, where specialized, and are mainly composed of ion channels and receptor proteins. Regarding neurons and glial cells as distributed parallel information processing units, their natural responsivity to various electric stimulus frequencies and chemicals in the membrane structure and aqueous media is performed by protein structure regulation. Interfacing directly with living networks is more than signal acquisition and feedback. Functional membrane structure regulation is also required *via* biochemical signaling activity.

The brain–machine interfaces can intervene in cellular signaling by influencing ion flow across membranes, and the membrane structures of brain cells are rich in function. Neurons and glial cells are just circuit boards or skeletons; specific protein structures are the logic elements on the membrane, such as synaptic structure, receptors, and ion channels. Liquid-ordered microdomains with proteins and lipids called lipid rafts are the basic units, the targets of signal acquisition and intervention, and the smallest regions performing computational functions. Glial cells regulate neuronal membrane proteins through secretion and lipid raft region of glial cells themselves, and neuroglial interactions may also be involved in nervous system functions. These channels, receptors, and enzyme proteins could interact with electrodes. For example, redox enzymes can achieve electronic communication with conductive electrodes *via* electrical, electrochemical, electron transfer mediators cyclic, and direct electron transfer from an enzyme active center to an electrode, and current is generated (Bollella and Katz, 2020).

On the basis of the proximity modulation of the membrane structure, the simulated ion circuit can utilize microfluidic planes of electrolytes in planar and hydrogels, graphene, and hexagonal boron nitride (Hou and Hou, 2021), based on the use of gradients generated by the selective permeability of biological membranes. In biological systems, ions are charge carriers. The action potential across cell membranes is to transmit data based on the time and voltage variant. Thus, the ionic conductivity modulation of the ion environment concludes in nanofluidic iontronics (Yang et al., 2019). Different ion conduction nanofluidic devices not only have signals compatible with neurons but also have working media compatible with the physiological aqueous environment. In natural organisms, ion channels have different structures and shapes. The asymmetric design of geometry and inner surface charge distribution in one-dimensional nanoconfined space can reproduce diode-like ion rectification properties in solution systems. In addition, the biological nanometer channel is dynamical to deformation, the curvature, and adjustable carbon nanotubes for dynamic real-time control of the ion rectifier. In addition, two-dimensional nanomaterials, such as graphene, boron nitride, and molybdenum disulfide, and the emergence of the enlarged translational degree of freedom of ion transport lead to increased interaction between ions, and the interaction forms are more diverse. Moreover, the lag effect of ion motion brings a potential memory effect to the ion circuit, which brings hope for the development and application of brain–computer interface and brain-like computing technology.

The function of the membrane with nanoscale structures can be achieved by proteins and chemical modulation, nanoparticles, *etc.* In addition to complexing in matrices such as artificial hydrogels and thus functioning, they can also naturally adsorb near neuronal and glial cell membrane structures. Therefore, penetrating the neural networks requires surface modifications to bind with nanocarriers that can target neurons and glial membrane or pass through the nanoscale glial blood –brain barrier. The nanocarriers include liposomes, lipid nanoparticles, polymeric particles, micelles, and dendritic molecules, as well as biological vectors such as gene transfer viruses, virus-like particles, and exosomes. For example, adenoviruses target neuronal, astroglia and glioma cells, and their transgene expression lasts for years in the peripheral tissues of primates. They can manufacture but triggers an immune response in the body (Terstappen et al., 2021). Therefore, virus-like particles might be a better choice for controllability and reduced immune response by mimicking neurological latent viruses. In addition, microglia can also be modified by optogenetics to influence chronic pain (Fyfe, 2021), and even improve BCI inflammation state and function by transferring the modulation message. This technique is also frequently used in neuronal manipulation, and in addition inflammation of glial cells will be discussed later. Furthermore, with the acceleration of semiconductor and nanotechnology, such wireless and injectable technics make defined cells manipulation in the deep brain less invasive and removable. In the next part, we will introduce the neuron structure and nanoparticle interfaces in detail.

## Neuron structure and nanoparticle interfaces

In most cases, a neuron has a long axon, which transmits electrical signals to other neurons, muscle fibers, or gland cells. Long axons, extensive dendrites, and varying synapses are constantly building up and degenerate. Nerve axons were cables that were wrapped with lipid glial cells and worked as natural output data lines that connect to the outside of the body. Nano-brain-computer interfaces resemble nerve fibers that build artificial membrane synapses and regeneration of axons. Virus-like nanoparticles can be designed to target neuron populations and accurately mediate with optical, magnetic, ultrasonic, chemical, and electrical signals.

Nano-brain-computer interfaces and virus-like nanoparticles are important for neuromodulations. The nanoscale array can directly form junctions with sub-neuron structure, while such accuracy can also be achieved with noninvasive nano modulation. For example, photon absorption elicits conformational changes in membrane proteins that switch ion channels’ selectivity, and then comes electrical current. It made precise and versatile neuronal selective manipulation with high resolution in time and space possible to modulate in both excitatory and inhibitory cells *in vivo* within living experimental individuals (Deisseroth, 2015). Since upconversion nanoparticles can absorb certain light of high penetrability at the wavelength of near-infrared and emit visible rays, transcranial near-infrared rays and modified upconversion nanoparticles can mediate optogenetics, initiating the release of dopamine from genetically tagged neurons in an experiment (Chen et al., 2018). And other well-penetrating forms of energy and information such as ultrasound and magnetic fields that can penetrate deeply are converted into physiologically regulated forms of information to achieve noninvasive and precisely combined brain–computer interaction. Such as magnetoelectric nanoparticles, the focused ultrasound that uncage neuromodulatory drugs, and nanoscale piezoelectric materials that transfer acoustic waves into electric fields (Yang et al., 2021). Moreover, attenuated viral or virus-like particles can conduct, for example, photosensitive channel genes, carry nanoparticles to targeted neural populations for manipulation, decipher neuronal connectivity, and perform functional analyses to enable neural information acquisition. Brain–computer interfaces with nanostructures require double-layer membranes and functional protein-coated RNA, *etc.*, which can be modified to target intracranial cells and improve blood–brain permeability. Sensory and motor neurons that extend beyond the skull are the entry point for viruses. The rabies virus spreads from presynaptic neurons to the starter neuron (Vogt, 2019). After entry, the viruses use the axonal transport system for retrograde transport to the brain. Along the axons that cross the blood–brain barrier (BBB), there are glial blood–brain barrier, such as receptor-mediated transcytosis and the use of neurotropic viruses, nanoparticles, and exosomes. To simulate the transport of rabies virus along the axoplasm *via* the natural nerve cell structure, it can be targeted to the neural nucleus by nanoparticle or viral vector; such device delivery requires biomedical materials to achieve. And with the surface-modified APOE peptides, they could target glial cells through the blood–brain barrier *via* LDLR receptors. By actively targeting molecules, adsorption, magnetic and ultrasound mediated trans-BBB can be developed by mimicking bioprocess and using artificially intelligent protein design tools, such as the RVG29 peptide inspired from rabies virus can act on nicotinic acetylcholine receptors to the brain. Surface chemistry, such as electric charge, and chemical groups affect the absorption route into the cell. Targeting ligands, such as antibodies, proteins, nucleic acids, or small molecules, can increase the targeting of the BBB (Terstappen et al., 2021).

In conclusion, targeted nanoparticles, viroid particles, RNA delivery techniques, and exosomes are directly implanted or injected into the brain alone or with BCI. Multifunctional soft substances can be used to promote neuron development and growth *via* various ways such as long-term electrical stimulation, drug-releasing, and scaffold building (Feng et al., 2015). Axons may be possible to regulate gene expression of controlled ion channels or wireless signaling as a signal transfer interface in the brain. Whether using translational nanomaterials, virus-like particles, enzymes or proteins, or photons, they are all means of intervening in neurological interventions that have different interface requirements and therefore need to be examined in practice according to the characteristics of each technology itself.

## Brain–computer interface inflammatory response and glial cells

The state of the BCI inflammatory response or the treatment of nervous system diseases is based on regulation of the glial and neuronal state regulation. The glial cells of the neural network affect specific pathways during chronic emotional states or sudden stimulation, and further functional sensitization or degeneration following occurs (Brites and Fernandes, 2015). It was expressed as upregulated astroglial potassium and glycogen synthase kinase 3 and upstream wingless-frizzled signaling pathways (Duman and Voleti, 2012; Cui et al., 2018).

While neurons and their circuits are almost considered to be the basic computational units, glial cells cannot be ignored. They not only have simple protective and nutritional functions, but they are functional partners of neurons. Abnormal glial cell states correspond to synaptic and electrical neuronal excitability (Venkatesh et al., 2019). Astrocytes and microglia play primary roles in synaptic shaping, secreting neuromodulators, ion homeostasis, and the formation of neural networks. To be specific, axon-wrapping glial cells surround axons, with insulating properties, which is due to the high content of lipids in myelin. What’s more, with the closed protection of the meninges and blood–brain barrier, the nervous system, and immune system have been previously considered separate while they practically work together to maintain brain health. As a matter of fact, rather than described as displaying immune privilege in the central nervous system (CNS), immunological surveillance and response are frequently carried out, as well as shape the neural network, mostly by microglia, the CNS residents, which have been shown for be essential to homeostasis, development, and neuroinflammation through communications with almost all components, including neurons and glia (Graykowski and Cudaback, 2021). Refined dynamic and localized changes between glial neuro have well cooperated. The microregion of glial cells has a variety of functions in accompanying its local neuronal environment, forming certain structures such as glial processes and dendritic spines. When an injury such as implanted invasive BCI occurs, astrocytes and microglia will form an inflammatory barrier and promote the development of related circuits for sensitive or inhibitory functions (Kofuji and Araque, 2021).

Signals between neurons and glia can significantly seize CNS. Activated by persistent psychosocial or oxidative stressors, a neuronal chronic inflammatory state occurs with synaptic and electrical changes. During the damage process, proliferated microglia are activated to help restore the brain environment, and normally their vast projections constantly surveil and scavenge the brain environment (Graykowski and Cudaback, 2021). However, activated microglia can secrete noxious signals, such as pro-inflammatory cytokines, reactive oxygen species, nitrogen species, and excitatory neurotransmitters, which further aggravate damage. The peripheral inflammatory state mutually promotes neural inflammation, which helps sensitize the CNS to a noxious stimulus. For example, CD11c+ microglia expressed insulin-like growth factor-1 increased when nerve hypersensitivity occurs after an injury; this type of cells and substances help reduce nervous hypersensitivity (Kohno et al., 2022). In addition to releasing neurotransmitters, neurons also release neuromodulators, and the substance and their receptors appear in neurons and glial cells (Rostene et al., 2007), that link and affect their environment. Based on this process, channel proteins that noted expressed in glial cells are actively involved in the dynamic neuroglial interactions (Giaume et al., 2021), such as connexins and gap junction channels. In this case, nanotubes in BCI could play a role as an external control switch that can be designed to regulate glia–glia and neuron–glia interactions. Thus, nanoscale and surface molecular modulations in BCI can be applied to regulate the recovery pathway, glia population differentiation, and further help neuron reconstruction.

## Feedback learning mechanism and exploration of network links

A whole BCI system works in the following steps, neuronal signal acquisition, data analysis, and feature extraction by machine learning, then translating to the neural network signals as output from the interface. Machine learning is the key to human–machine signal conversion. Instead of trying to understand the meaning and logic of neural activity patterns, machine learning greatly improves the ability to decode neural activity by identifying and linking the patterns to the user’s intent in the face of the problem. For paraplegic patients with spinal cord or motor neuron injury, the motor intention still exists in the brain network after paralysis. By recording neural activity from microelectrode arrays in the premotor area in the cortex while the participant imagines doing a specific activity. Artificial intelligence immediately decodes motor intentions represented by neural activity in the motor cortex and makes it into actual behavior (Willett et al., 2021). However, the information comes from the instantaneous potential changes on a two-dimensional surface, so it is limited to understanding the deeper network functions such as logical thinking and memory.

**FIGURE 2:**
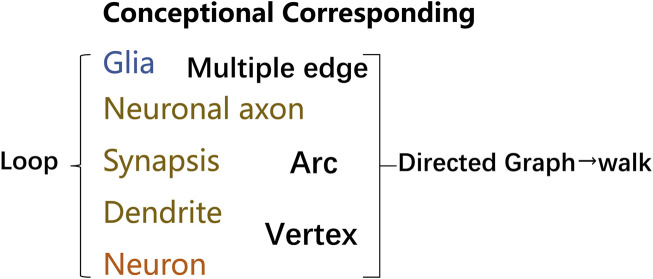
Terminology in graph theory corresponding to neural networks. Graphs provide a better way to deal with abstract concepts such as relationships and interactions. Neural loops can be abstracted into graphs utilizing concepts, and graphs made up of vertex and edge may be described analytically or as matrices. Glial cells can be represented as nodes or edges, many of which can correspond to multiple edges, where edges can be directed vectors or arcs. Similarly, dendrites and neurons can be represented as vertex, with edges and nodes labeled with weights that reflect varying degrees of information in positive or negative directions. Neuronal axons can be used as arcs to link different related functional regions, and neural loops can be formed between many neuronal structures. A walk is a finite sequence that takes the form of a graph’s vertices and edges. The walk is open if the beginning and last vertices are different. The walk is a closed loop if the initial and final vertex is the same.

To further describe the networks, simplified tools for describing net and data structure are seen as essential. Limited to the skull space constraint, unique features along the lines of efficient topology network communication and information processing. And knowing the characteristics of basic elements, modeling networks, and simplifying by mathematical methods can be critical to understanding the networks and how they function mentally. Therefore, a language that clearly describes the structure in neural networks is of urgent need. Graph theory was introduced, which is defined as sets of nodes and edges that represent system elements and their interrelations. Language systems demonstrate the properties of networks, geometry, and spatial anatomical connectivity in the context of structural constraints. The correlated edges in graph theory can indicate the functional correlation between neuronal groups in addition to the neural-directed connections. As shown in Figure 2, through imaging, functional experiments and artificial intelligence analysis, the connection of a neural network can be expressed by the corresponding concepts in graph theory.

**FIGURE 3:**
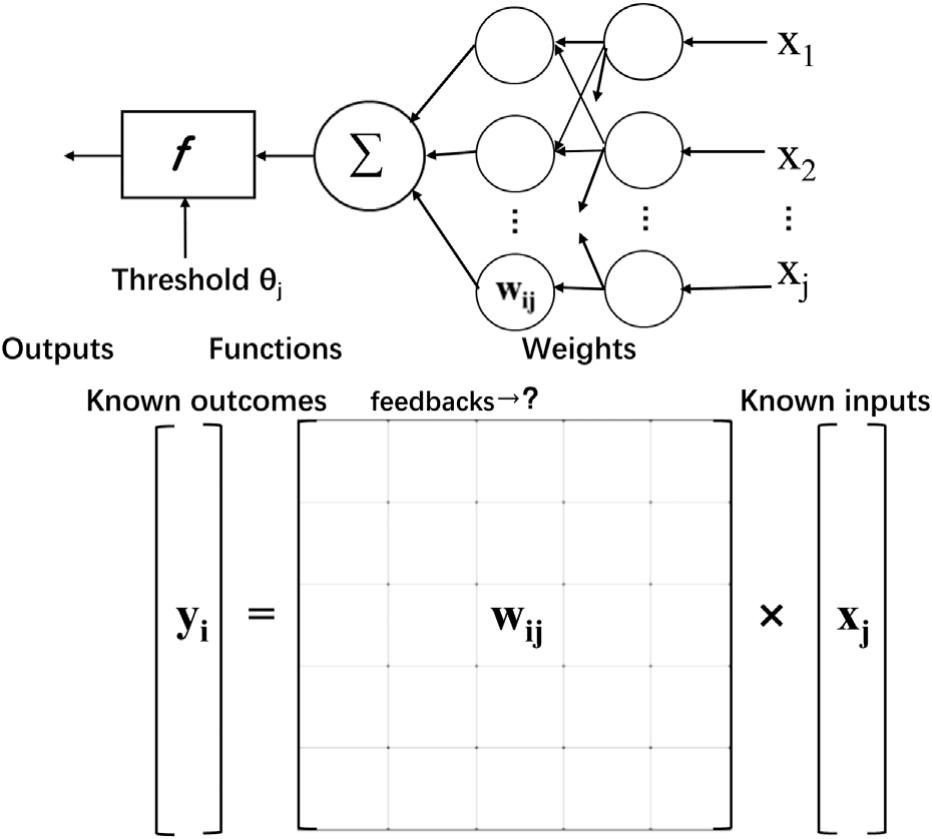
Simplified feedback learning process of calculating the unknown neuron value. Similar to solving equations in a matrix with known inputs and outputs, corresponding to received information and learned judgment and behavioral control information, the neural network derives parameter processing information, that is, weights, through feedback.

In addition, the breakthrough in computer science also provides a new approach to modeling nervous networks. Advances have been made in the application of artificial intelligence, which can sort objects out of raw data by computing, that is, to extract meaning and partly supervised learning. The function that seeks to realize a certain purpose can be visualized in Figure 3. However, the nervous system, especially the human brain, is far more complicated than a single model can convey, especially the dynamic integration on different scales.

Bionic artificial neural network’s logical correspondence makes the implementation in brain biomedical interfaces accessible. Signal input and processing breakthroughs are also inspired by mechanisms of brain-like feedback learning. Technically, the brain’s information processing ability benefits from the massive parallel and distributed computing model, which means thinking and memory happened simultaneously. In contrast, the computer is composed of independent and fixed CPUs and storage units. Inspired by neural networks, Artificial Neural Network has generated and solved many practical problems. The model is composed of many layers of nodes, also called neurons, connected by tied connections. Each node represents a specific activation function and the connections between nodes are given weighted values *via* feedback. It is an equation solving or function approximation process. By learning about weight through enormous feedback, computers are trained with professional recognition and translation skills. Machines can easily learn to simulate the language with this technic, helping to better shape the interface with functional communication. With the hope of building a hardware neural network interface, attempts have been made to create artificial neural network algorithms.

Once the machine learned the neuronal codes, part of the language spoken by neurons can be programmed and in turn, translated into specific meaning. Now the challenge lies in brain–computer Interface that breaks and expands the physical net limits. Brain–computer interfaces remarkably restore the ability to speak and movement control by training an artificial neural network, which transforms the neural activity into probabilities data of movement intentions.

## Conclusion

For invasive BCI systems, good biocompatibility is needed, making it more valuable for us to learn, adapt, and recover various forms of sensory and motor control. Thereby, for example, sensory transduction, control of robotic arms and computers, and even driving (Aflalo et al., 2015) can be reliable throughout the lifetime. In addition, the richness of human sensory forms such as pressure, temperature, vibration, and light form the feeling of touch, warmth, hearing, and vision, which implies a multitude of possibilities for the conversion of signals into neural electrical impulses. And ultrasound, cables, near-infrared light, nano-temperature-controlled channels, and magnetism can all be used as forms of propagating information into the skull. From a functional point of view, brain–computer interface systems can be divided into input for various sensing functions such as somatosensory and special senses such as hearing and vision, and generate motor control such as communication, restoration of hand function, modulation of the autonomic nervous system and even memory and learning, and participation in a variety of functions in the consciousness process. This requires precise control of brain areas and adequate connection to neural networks, forming a similar afferent–transmitter interface between the spinal cord and the brain nerves, where the brain and machine signals are processed, continuously adapted and learned through feedback.

Glial cells play a role in dynamic changes that are related to neuronal function and state, it was important neuronal wiring in the network of neurons and glia, certain information is learned and processed through certain cellular connection structures of neural pathways or the same neurotransmitters, therefore performing specific functions in specialized regions of the complex network. Where neuronal connections depend on information and feedback to survive and establish. This process can be facilitated by a local electrical signals or chemical substances signals but also by optical, acoustical, and magnetic signals through genetic strategy or nanotechnology. In short, with the regulation of glial cells, electronic impulses carrying information, and even accompanied by gene transfer, an efficient interface system may be cultivated. Furthermore, there are many perspectives on the design of the brain–computer interface. If more interfaces are applied to better fit the brain’s anatomical structure, currently only a local plane signal, a dynamic and multi-dimensional information network of neurons and glia, the cellular or molecular scale will approach, and more nanoparticle- based empirical models will simplify and create intelligent networks.

After fully interpreting these five-part processes, brain–computer interface technology enables the transformation of signal and regulation from glial cell–mediated nanomaterial to further fit the biological neural network and achieve minimally invasive and higher signal quality from signal relays such as photons, magnetism, and ultrasound to realize the regulation and supplement of brain function. The understanding of the natural structure of the brain can promote optimization of the design and modification and adjustment of functional groups, realize the opening and closing, control and adjustment of ion channels, and clear the operation mode of the bain–computer interface in a stable and accurate brain–computer interaction and self-learning through feedback.
